# Acute kidney injury in hospitalized patients with nonexacerbated chronic obstructive pulmonary disease

**DOI:** 10.1186/s12890-020-1161-7

**Published:** 2020-04-29

**Authors:** Xiaohong Wang, Zhen Xie, Shuguang Xiong, Wei Xiong, Tian Zhong, Yang Su

**Affiliations:** 1 Department of Respiratory Medicine, the Second Affiliated Hospital of Chengdu Medical College, Nuclear Industry 416 Hospital, Chengdu, China; 2 Department of Dermatology, Sichuan Provincial People’s Hospital, University of Electronic Science and Technology of China, Chengdu, China; 3 Chinese Academy of Sciences Sichuan Translational Medicine Research Hospital, 32 West Second Section, First Ring Road, Chengdu, 610031 China; 4 Division of Urology and Organ Transplantation, Department of Surgery, Sichuan Provincial People’s Hospital, University of Electronic Science and Technology of China, Chengdu, China; 5 Department of Respiratory Medicine, Sichuan Provincial People’s Hospital, University of Electronic Science and Technology of China, Chengdu, China; 6 Clinical Laboratory, Sichuan Provincial People’s Hospital, University of Electronic Science and Technology of China, Chengdu, China

**Keywords:** Nonexacerbated chronic obstructive pulmonary disease, Acute kidney injury, Epidemiological study, Risk factor, Clinical burden, In-hospital mortality

## Abstract

**Background:**

The epidemiology of acute kidney injury (AKI) in nonexacerbated chronic obstructive pulmonary disease (NECOPD) patients is unknown. This study investigated the factors associated with AKI and the association between AKI and in-hospital mortality in the hospitalized NECOPD population.

**Methods:**

The electronic medical records of 2897 patients hospitalized with NECOPD were analyzed retrospectively. Demographic information, medicine used before AKI, diagnosis records and laboratory data were collected. AKI was classified as community-acquired (CA-) or hospital-acquired (HA-) AKI according to the serum creatinine criteria. Risk factors for HA-AKI and in-hospital mortality were analyzed by logistic regression analyses. To avoid an interaction between cor pulmonale and AKI, the association between AKI and in-hospital morality was further analyzed with cor pulmonale stratification.

**Results:**

The incidence rates of CA- and HA-AKI were 7.1 and 12.0%, respectively. Increased age, female sex, cor pulmonale comorbidity, chronic kidney disease stage, diuretic and glycopeptide use before AKI and iodine-containing contrast medium exposure were independently associated with HA-AKI. A total of 5.7% of the patients died. After adjustment for age, sex, cor pulmonale, chronic kidney disease, Charlson comorbidity index score (without renal disease) and hemoglobin level, HA-AKI was an independent risk factor for in-hospital mortality [OR 13.909 (95% CI 8.699–22.238) in non-cor pulmonale subgroup; OR 26.604 (95% CI 12.166–58.176) in cor pulmonale subgroup], whereas CA-AKI was not.

**Conclusions:**

AKI is common in the NECOPD population. Diuretics and contrast media are associated with HA-AKI in this population. The patients with HA-AKI have a higher mortality risk than the patients without AKI.

## Background

Chronic obstructive pulmonary disease (COPD) is characterized by chronic airflow limitation, inflammation and lung remodeling. It is a progressive disease that results in a significant burden, both medically and financially. COPD is the third leading cause of death among diseases, resulting in a great economic burdens to society [1]. Acute exacerbations of COPD (AECOPD) require emergent medical care, and their estimated 3-year all-cause mortality rates are as high as 50% in patients who require hospitalization [2]. In addition to urgent therapy for AECOPD, care under stable conditions is also essential for COPD patients. It is worth noting that many hospitalized patients admitted to respiratory departments or other units have nonexacerbated COPD (NECOPD). Even without respiratory infection, the patients with NECOPD had a relatively higher risk of mortality [3]. Hypoxemia is an elementary pathophysiological disorder associated with COPD, regardless of acute or stable conditions. Due to the systemic damage of hypoxemia, the recognition of extrapulmonary complications will facilitate effective personal management.

The lung interacts with the kidney in the body [4]. Acute kidney injury (AKI) is a common syndrome in clinical practice. According to a preliminary analysis of 266 studies worldwide, 21% of hospitalized patients presented AKI [5]. AKI is closely associated with a high clinical burden and poor prognosis, such as in-hospital mortality, and therefore is considered a high-risk complication. Previous studies have shown that AKI increases in-hospital mortality in patients with AECOPD [6, 7]. Fabbian et al. [6] reported a 5% AKI incidence in 7073 patients. They also demonstrated that AKI was associated with a 284.9% higher mortality risk. They used the International Classification of Diseases codes, which were specific but had low sensitivity, as an AKI diagnosis standard [8]. Recently, Cao et al. [7] reported an overall AKI prevalence of 21.3% in the AECOPD population using the Kidney Disease: Improving Global Outcomes (KDIGO) criteria. Notably, although all the abovementioned studies investigated the risk factors of AKI, they did not consider the influence of drug use.

Compared with AECOPD, NECOPD has different pathophysiological disturbances and clinical features. Patients with AECOPD are hospitalized mainly in a respiratory department or intensive care unit, as a high proportion of patients require pulmonary support, whereas NECOPD patients may be admitted to other divisions. Furthermore, the complications, medications and diagnostic techniques for AECOPD and NECOPD differ slightly. Although NECOPD has been included as a variable in the analysis of risk factors, such as the Cleveland scoring system to predict AKI after cardiac surgery for AKI [9], the epidemiology of AKI in patients with NECOPD has not yet been investigated.

In this study, we investigated the factors associated with AKI and the association between AKI and in-hospital mortality in the hospitalized NECOPD population.

## Methods

### Study patients

The current study retrospectively analyzed the clinical data of adult COPD patients (≥18 years) who received treatment at Sichuan Provincial People’s Hospital between January 2011 and September 2016. All the patients with COPD discharge diagnoses were selected. The patients with a diagnosis of AECOPD or any comorbid respiratory infection were considered to have AECOPD, while the others were considered to have NECOPD. The exclusion criteria were as follows: 1) the patient’s baseline serum creatinine levels were unavailable; 2) the patient had advanced chronic kidney disease (CKD), i.e., an estimated glomerular filtration rate (eGFR) < 15 ml/min/1.73 m^2^; a minimum SCr level > 353.6 μmol/L (4 mg/dL) during the hospital stay; a clinical diagnosis of stage 5 CKD; or the receipt of a replacement kidney before the onset of AKI; 3) the patient had a maximum SCr level < 53 μmol/L during the hospital stay (to avoid an AKI diagnosis due to a very slight SCr increase and potential malnutrition); 4) the patient previously underwent kidney transplantation or amputation; and 5) the patient had incomplete clinical records that were unable to supply the data for the current research.

Based on the clinical records, medical costs, lengths of hospital stay and in-hospital mortality rates associated with AKI were analyzed as medical burdens; the main outcome was in-hospital mortality.

### Observational indices

AKI was diagnosed according to the criteria provided by the Kidney Disease: Improving Global Outcomes (KDIGO) guidelines; AKI was confirmed if the SCr value increased by more than 50% compared to the baseline value within 7 days [10]. Considering that the SCr tests before admission are frequently not available, the lowest creatinine level from 1 month before admission to the time of discharge was regarded as the baseline for community-acquired (CA-) AKI. Patients admitted to the hospital with AKI based on a creatinine measurement within 48 h of hospital admission were considered to have CA-AKI [11]; the other patients were considered to have hospital-acquired (HA-) AKI [10]. CKD was classified based on the eGFR, which was calculated by the minimal SCr value within 1 year, according to the EPI equation of the Chronic Kidney Disease Epidemiology Collaboration [12].

Complications were adjusted for based on discharge diagnoses, and the Charlson comorbidity index (CCI) score was evaluated (the CCI is a scoring system that assigns weights to mortality-related comorbid conditions, and it involves 19 comorbidities, namely, myocardial infarction, congestive heart failure, peripheral disease, cerebrovascular disease, chronic pulmonary disease, dementia, connective tissue disease, peptic ulcer disease, mild liver disease, diabetes without end-organ damage, hemiplegia, mild to moderate renal disease, diabetes with end-organ damage, tumor without metastasis, leukemia, lymphoma, moderate or severe liver disease and metastatic solid tumor) [13]. The total CCI score was used as the primary index for predicting in-hospital mortality [14]. The renal disease item in the CCI was assessed based on either a corresponding diagnosis of moderate to severe renal disease or an eGFR < 45 ml/min/1.73m^2^. We also calculated the CCI score after removing renal disease to avoid potential collinearity between CKD and CCI when they were both included as variables in one multivariable analysis. Anemia was defined as a minimum hemoglobin (Hb) concentration < 120 g/L during hospitalization, regardless of sex, according to the World Health Organization standard, because amenorrhea data was unavailable in the female subjects. The Hb level was stratified as < 6 g/dL, 6–8.9 g/dL, 9–11.9 g/dL and ≥ 12 g/dL.

### Statistical analyses

Measurement data are presented as the median [25th quartile (Q25)-75th quartile Q75)], and independent Mann-Whitney-U tests were performed for group comparisons. Enumeration data are presented as case numbers, and *χ*^2^ tests were used for the comparisons. Multiple comparisons were corrected using the Bonferroni method. Univariate logistic regression was performed, and the variables with a statistically significant difference were included in the multivariate logistic regression analysis by a stepwise forward conditional model. Odds ratios (ORs) and 95% confidence intervals (CIs) were calculated. All statistical analyses were performed with SPSS 24.0, and a bilateral *P* value < 0.05 was considered statistically significant.

## Results

### General data

**Fig. 1:**
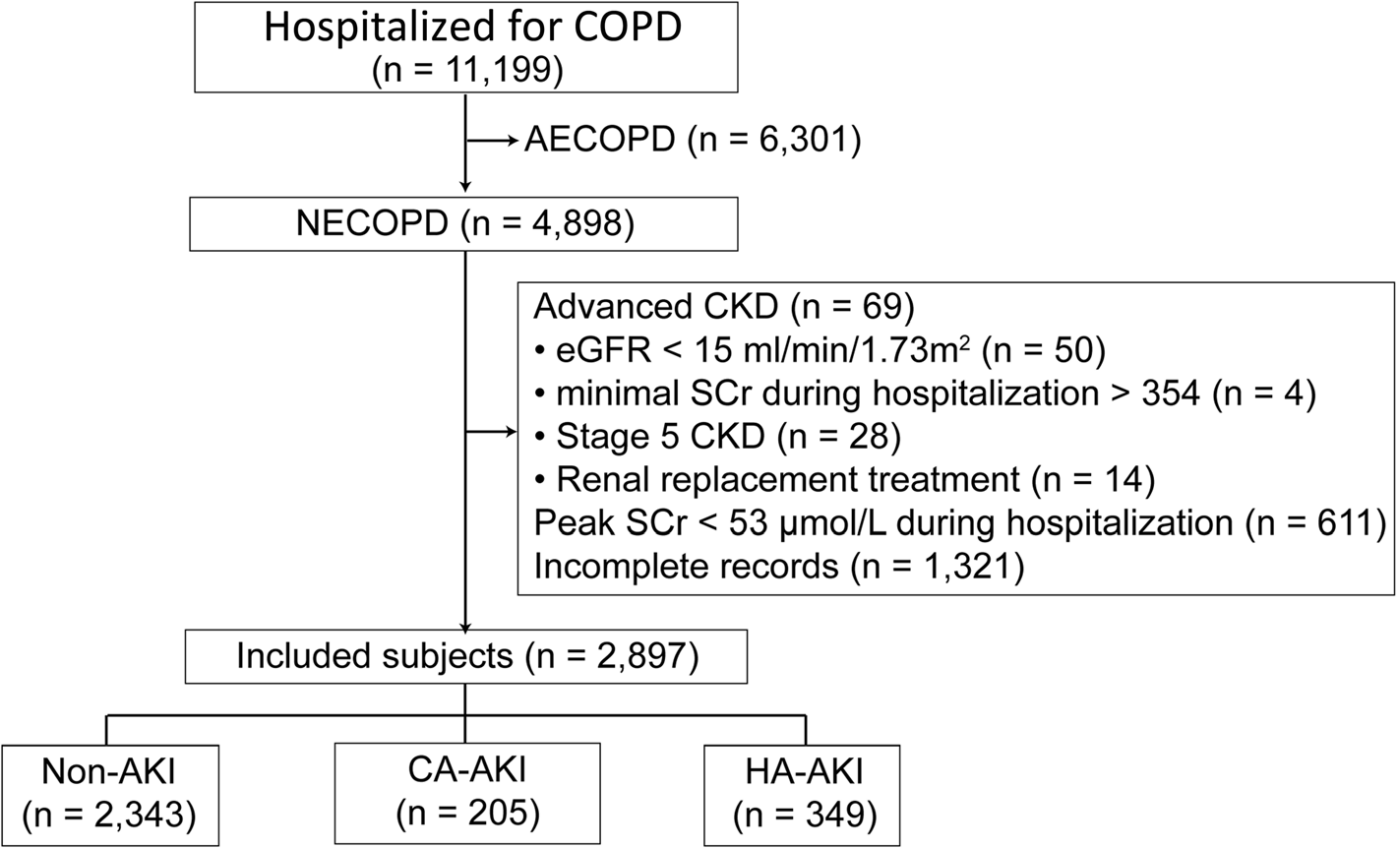
Inclusion flow chart. COPD: chronic obstructive pulmonary disease; NECOPD: nonexacerbated COPD; CKD: chronic kidney disease; eGFR: estimated glomerular filtration rate; SCr: serum creatinine; AKI: acute kidney injury; CA: community-acquired; HA: hospital-acquired

A total of 11,199 patients hospitalized for COPD were investigated during this study, 4898 patients were nonacute. After applying the exclusion criteria, this study enrolled 2897 NECOPD patients. Compared with the excluded patients, those enrolled were 1 year older (75 vs 74, *P* = 0.003), more often male (77.9% vs 67.2%, *P* < 0.001), less often had cor pulmonale (22.8% vs 55.9%, *P* < 0.001), and had an eGFR that was 4.7 ml/min/1.73m^2^ lower (91.7 vs 87.0, *P* < 0.001). Among these patients, 205 patients (7.1%) had CA-AKI, and 349 patients (12.0%) had HA-AKI (Fig. 1). In the CA-AKI and HA-AKI groups, 62 (9.4%) and 113 (17.1%) patients, respectively, presented with cor pulmonale complications. The general data of the patients with the different types of AKI are summarized in Table 1.

**Table 1:** The basic clinical characteristics of the study patients

	Non-AKI	CA-AKI	HA-AKI	*P* value
Age, year	75 (67, 82)	74 (66, 82)	78 (72, 84)	< 0.001
Male, *n* (%)	1852 (79.0%)	158 (77.1%)	247 (70.8%)	0.002
Cor pulmonale comorbidity, *n* (%)	486 (20.7%)	62 (30.2%)	113 (32.4%)	< 0.001
Admission department, *n* (%)				< 0.001
Respiratory	576 (24.6%)	54 (26.3%)	82 (23.5%)	
Intensive care	134 (5.7%)	21 (10.2%)	44 (12.6%)	
Cardiovascular	426 (18.2%)	27 (13.2%)	80 (22.9%)	
Other	1207 (51.5%)	103 (50.2%)	143 (41.0%)	
Maximal SCr (μmol/L)	92.4 ± 31.4	115.0 ± 57.6	176.3 ± 117.4	< 0.001
Minimal SCr (μmol/L)	78.4 ± 25.9	67.5 ± 25.4	88.4 ± 46.2	< 0.001
eGFR (ml/min/1.73 m^2^)	87.1 ± 18.3	96.6 ± 23.6	80.2 ± 27.8	< 0.001
Anemia, *n* (%)	1173 (50.1%)	135 (65.9%)	267 (76.5%)	< 0.001
CCI	5 (3, 6)	5 (3, 7)	5 (4, 8)	0.148

*SCr* serum creatinine; *eGFR* estimated glomerular filtration rate; *CCI* Charlson comorbidity index

### Factors associated with HA-AKI

Due to a lack of preadmission data for the CA-AKI group, only the associations between clinical data and the development of HA-AKI were analyzed in this study. As medication concerned sensitive data, only drugs that are commonly used for diseases of respiratory origin with potential renal toxicity were retrieved in this study. The prescription time and AKI occurrence were analyzed, and only the medicines used before AKI were further studied. These drugs included proton pump inhibitors (use prevalence, 55.0%), diuretics (including osmotic diuretics such as mannitol; use prevalence, 28.6%), rennin-angiotensin system inhibitors (use prevalence, 26.7%), nonsteroidal anti-inflammatory drugs (NSAIDs; use prevalence, 18.8%), intravenous diodone (use prevalence, 12.2%), intravenous antihemorrhagic drugs (use prevalence, 10.0%), aminoglycosides (use prevalence, 4.8%) and glycopeptides (use prevalence, 2.4%). Univariate regression showed that increased age, female gender, cor pulmonale complication, CKD and diabetes were associated with HA-AKI. Among the drugs with a use prevalence greater than 10%, diuretics and intravenous diodones used before AKI were associated with HA-AKI. Despite a low use prevalence, glycopeptides were associated with the development of HA-AKI (Table 2). The variables with *P* values < 0.10, i.e., age > 65 years, female gender, diabetes complication, cor pulmonale complication, CKD stage, and pre-AKI diuretic, diodone and glycopeptide use, were included in the multivariate model, according to the univariate regression analyses (Table 2).

**Table 2:** Analysis of the risk factors for HA-AKI

	Univariate model			Multivariate model		
	OR	95% CI	*P* value	OR	95% CI	*P* value
Increased age	1.899	1.324–2.724	0.001	1.463	1.003–2.134	0.048
Female	1.558	1.212–2.002	0.001	1.498	1.149–1.952	0.003
Cor pulmonale	1.830	1.431–2.339	< 0.001	1.790	1.379–2.324	< 0.001
CKD			< 0.001			< 0.001
Stage 1–2	Reference			Reference		
Stage 3a	1.775	1.163–2.708	0.008	1.754	1.136–2.709	0.011
Stage 3b	3.139	1.862–5.292	< 0.001	3.161	1.850–5.401	< 0.001
Stage 4	6.478	3.532–11.882	< 0.001	6.575	3.531–12.243	< 0.001
Diabetes	1.357	1.045–1.762	0.022	Not entered in final model		
Clinical medication						
Proton pump inhibitors	1.142	0.910–1.434	0.253	Not selected		
Diuretics/dehydrants	1.950	1.547–2.459	< 0.001	1.527	1.193–1.954	0.001
RAS inhibitors	1.081	0.841–1.389	0.544	Not selected		
NSAIDs	0.967	0.723–1.293	0.821	Not selected		
Intravenous diodones	1.928	1.436–2.588	< 0.001	2.048	1.499–2.798	< 0.001
Intravenous hemostatics	0.927	0.632–1.359	0.697	Not selected		
Aminoglycosides	1.256	0.769–2.053	0.362	Not selected		
Glycopeptides	4.141	2.469–6.944	< 0.001	3.991	2.322–6.859	< 0.001

*HA-AKI* hospital acquired-acute kidney injury; *CKD* chronic kidney disease; *RAS* renin-angiotensin system; *NSAIDs* nonsteroidal anti-inflammatory drugs

### AKI-related clinical burdens

The total hospitalization costs for the non-, CA- and HA-AKI groups were 3238 (2216, 5003), 4484 (3165, 7756) and 4759 (3028, 7481) US dollars, with daily costs of 220 (168, 312), 279 (205, 480) and 308 (213, 518) US dollars, respectively; the lengths of hospital stay for the three groups were 14 days (11, 20), 16 days (13, 21) and 16 days (13, 22), respectively (the data regarding the costs and hospital stays are all presented as the median (Q25, Q75); therefore, the median of the total hospitalization costs cannot be obtained by multiplying the median of the daily costs with that of the hospital stays in the corresponding group); there were significant differences between the AKI groups and non-AKI group (*P* < 0.001).

**Fig. 2:**
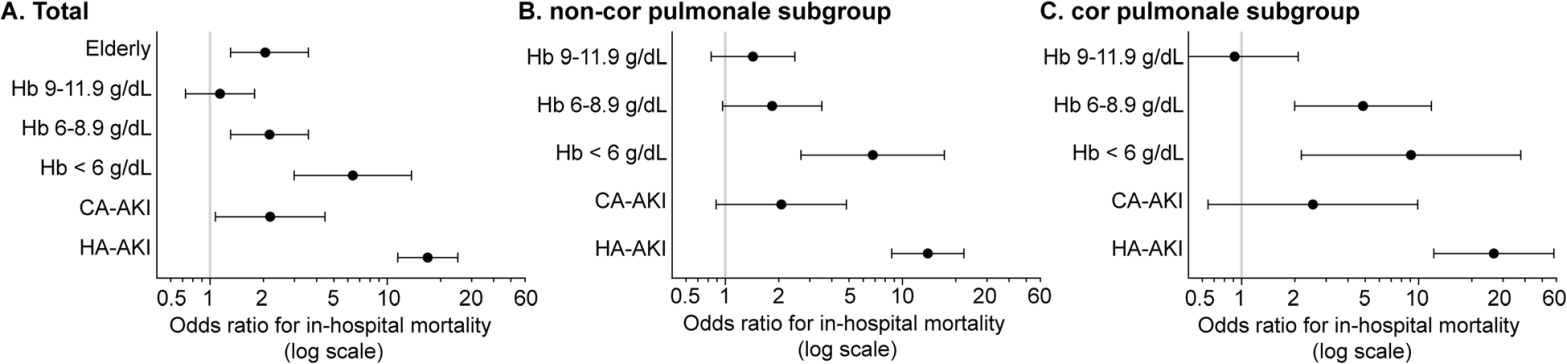
Association between AKI and in-hospital mortality. The variables included in the multivariate models were increased age (> 65 years), female sex, CCI, Hb level (minimum values during hospitalization stratified by ≥12, 9–11.9, 6–8.9 or < 6 g/dL) and AKI. The models included a total of 2897 patients (**a**), 2236 patients without cor pulmonale (**b**) and 661 patients with cor pulmonale (**c**). Hb: hemoglobin; CA-AKI: community-acquired acute kidney injury; HA-AKI: hospital-acquired acute kidney injury

A total of 164 patients (5.7%) died during hospitalization. Compared with the surviving patients, the nonsurviving patients were older, more likely be female and had higher comorbidity burdens (Table 3). The relation between AKI and in-hospital mortality was further analyzed. According to the univariate regression analysis, age > 65 years, female gender, cor pulmonale complication, CKD stage, CCI score without renal disease, Hb level (according to the minimum values during hospital stay, < 6 g/dL, 6–8.9 g/dL, 9–11.9 g/dL and ≥ 12 g/dL) and AKI type were associated with in-hospital mortality (Table 4). A multivariate logistic regression model was then constructed for further analysis. Because the CCI already considers CKD, the CCI score without renal disease but not the CCI score with renal disease was included in the model. In addition, due to the interaction between AKI and cor pulmonale according to the preliminary analysis, the cor pulmonale variable was excluded from the multivariate model. The results showed an independent association of in-hospital mortality with increased age [OR 2.051 (95% CI 1.305–3.591)], decreased Hb [OR 1.140 (95% CI 0.727–1.786) for 9–11.9 g/dL; OR 2.165 (95% CI 1.305–3.591) for 6–8.9 g/dL; OR 6.403 (95% CI 2.986–13.730) for < 6 g/dL] and AKI [OR 2.186 (95% CI 1.072–4.455) for CA-AKI; OR 16.949 (95% CI 11.483–25.016) for HA-AKI] (Fig. 2a). Then, we analyzed the independent relation between AKI and in-hospital mortality stratified by cor pulmonale. In both of the subgroups (with or without cor pulmonale), only HA-AKI was an independent risk factor for in-hospital mortality [OR 13.909 (95% CI 8.699–22.238) in the non-cor pulmonale subgroup; OR 26.604 (95% CI 12.166–58.176) in the cor pulmonale subgroup]; however, CA-AKI was not [OR 2.070 (95% CI 0.888–4.828) in the non-cor pulmonale subgroup; OR 2.532 (95% CI 0.647–9.910) in the cor pulmonale subgroup] (Fig. 2b and c).

**Table 3:** The basic clinical characteristics of surviving and nonsurviving patients

	Surviving	Nonsurviving	*P* value
Age, years	74 ± 10	79 ± 8	< 0.001
Male, *n* (%)	2142 (78.4%)	115 (70.1%)	0.013
Cor pulmonale comorbidity, *n* (%)	603 (22.1%)	58 (35.4%)	< 0.001
Admission department, *n* (%)			< 0.001
Respiratory	657 (24.0%)	55 (33.5%)	
Intensive care	167 (6.1%)	32 (19.5%)	
Cardiovascular	510 (18.7%)	23 (14.0%)	
Other	1399 (51.2%)	54 (32.9%)	
eGFR (ml/min/1.73 m^2^)	87.4 ± 19.9	80.3 ± 26.7	< 0.001
Anemia, *n* (%)	1447 (52.9%)	128 (78.0%)	< 0.001
CCI score	3 (2, 4)	4 (2, 8)	< 0.001

*SCr* serum creatinine; *eGFR* estimated glomerular filtration rate; *CCI* Charlson comorbidity index

**Table 4:** Analytical outcomes of AKI and in-hospital mortality according to the univariate model

	OR	95% confidence interval	*P*
Increased age	3.046	1.639–5.660	< 0.001
Female	1.544	1.092–2.184	0.014
Cor pulmonale	1.933	1.386–2.696	< 0.001
CKD			< 0.001
Stage 1–2	Reference		
Stage 3a	1.351	0.715–2.554	0.354
Stage 3b	2.382	1.165–4.872	0.017
Stage 4	6.834	3.450–13.539	< 0.001
Diabetes	1.170	0.805–1.702	0.411
CCI score	1.189	1.134–1.247	< 0.001
In-hospital minimum Hb			< 0.001
≥ 120 g/L	Reference		
90–119	2.134	1.419–3.210	< 0.001
60–89	5.456	3.486–8.538	< 0.001
30–59	10.058	5.205–19.436	< 0.001
AKI			
Non-AKI	Reference		
CA-AKI	2.505	1.246–5.035	0.010
HA-AKI	21.599	14.954–31.197	< 0.001

*CKD* chronic kidney disease; *CCI* Charlson comorbidity index; *Hb* hemoglobin; *AKI* acute kidney injury; *CA* community-acquired; *HA* hospital-acquired

## Discussion

In this first epidemiologic study in a hospitalized NECOPD population, we observed that 7.1 and 12.0% of patients had CA-AKI or HA-AKI, respectively, and the patients with cor pulmonale had a higher incidence of AKI. Increased age, female gender, cor pulmonale comorbidity, CKD stage, diuretic usage, exposure to contrast medium and glycopeptide antibiotic usage before AKI were associated with HA-AKI. After multiple adjustments for age, female gender, CCI and minimum Hb level, HA-AKI was an independent risk factor for in-hospital mortality, regardless of the presence or absence of cor pulmonale.

Before our study, Cao et al. reported that 15.8 and 5.5% of hospitalized Chinese patients with AECOPD had CA-AKI and HA-AKI, respectively [7]. The previously reported HA-AKI incidence was lower than that in our study. Theoretically, pathophysiological disturbance is worse in AECOPD than in NECOPD. The inconsistency between the two studies might be attributed to the much longer intervals for baseline SCr before admission for CA-AKI determination in Cao’s study (1 year) than in our study (1 month). An increased interval should cause a lower baseline creatinine level and result in an increased incidence of CA-AKI a reduced incidence of HA-AKI, as in Cao’s report. In addition, patients with extremely low SCr levels were excluded from our study (Fig. 1) but not in Cao’s study. Thus, the enrolled patients with HA-AKI in our study had higher creatinine levels (Table 1). Finally, the difference in clinical characteristics between the two studied hospitals might also be involved. In recent epidemiological data, the incidence of total AKI was 11.6% in hospitalized Chinese patients [11]. Regardless of the AKI type, our and Cao’s results together showed that COPD patients had a markedly higher prevalence of AKI than the general hospitalized population. Ours was the first report to reveal the AKI risk in nonexacerbated COPD, which indicates that NECOPD is not a stable condition from a renal perspective.

Previous studies have reported that AECOPD complicated with AKI increased in-hospital mortality [6, 15]. Our study showed that all types of AKI significantly increased the length of hospital stay and cost in the NECOPD population. After multivariate corrections, complicated HA-AKI remained associated with high in-hospital mortality. Although CA-AKI was correlated with an increased risk of in-hospital mortality according to the univariate logistic regression model and the model without the consideration of cor pulmonale, the lower OR limit of HA-AKI was higher than the upper OR limit of CA-AKI, suggesting that the mortality risk in HA-AKI patients was significantly higher than that in CA-AKI patients. After the patients were subdivided according to the presence of cor pulmonale, no further significant difference was observed. These results agreed with those of a previous study reported by Cao et al. that showed that the risk of in-hospital mortality in HA-AKI patients was significantly higher than that in CA-AKI patients [6].

In this study, all data used for analysis were from the electronic medical records of patients; this strength allowed us to classify the medications administered before AKI. The associations between the use of diuretics, intravenous diodone and glycopeptides and HA-AKI were not surprising; however, the high use prevalence rates above 10% for diuretics and intravenous diodones should be paid attention to in clinical practice. Another convenience of the use of electronic data was the availability of diagnostic and laboratory records. Thus we could weigh the mortality-related comorbidities, such as cor pulmonale, anemia, CKD and the CCI score. The diagnosis of renal disease is commonly missed in China [16]; therefore, we used a complex definition of moderate to severe kidney disease for the CCI score. In addition to the 230 patients diagnosed with moderate to severe kidney disease, 60 patients with an eGFR < 45 ml/min/1.73m^2^ were also identified.

Due to the retrospective design, this study failed to differentiate aspirin from other NSAIDs, which might contribute to the negative relationship between the use of NSAIDs, traditionally considered to be drugs with renal toxicity, and HA-AKI. Recent studies have indicated that a low dose of aspirin might protect against or at least not exacerbate AKI [17, 18]. Therefore, the effects of aspirin and other NSAIDs on AKI needs further evaluation. In addition, our study also had several other limitations. The pulmonary function and blood gas analysis indices were not available. Thus, the severity of hypoxia and the patient conditions could not be evaluated. In addition, approximately one-third of the patients were excluded due to incomplete medical records (Fig. 1), possibly leading to selection bias. Another limitation was that the only adverse outcome observed was in-hospital mortality, and other adverse events, such as quality of life, cardiovascular events and long-term survival, were not investigated.

## Conclusion

AKI is a common condition in the NECOPD population. Diuretics and contrast media are the frequently used medications associated with HA-AKI. Patients with HA-AKI have a higher mortality risk than those without AKI.

## Data Availability

All data used for this analysis were included in the article.

## Abbreviations

COPD: Chronic obstructive pulmonary disease;
AKI: Acute kidney injury;
NECOPD: Nonexacerbated chronic obstructive pulmonary disease;
CKD: Chronic kidney disease;
eGFR: Estimated glomerular filtration rate
